# Supplementary material for: Genomics-based approach for detection and characterization of SARS-CoV-2 co-infections and diverse viral populations
Source: Microbiol Spectr. 2025 May 1;13(6):e02092-24. doi: 10.1128/spectrum.02092-24 (PMC12131723; doi:10.1128/spectrum.02092-24)
Supplement: Table S1 — VOC-specific mutations and iSNV ratios present in confirmed SARS-CoV-2 co-infections. [file spectrum.02092-24-s0001.docx]

**Supplementary Table 1.** VOC-specific mutations and iSNV ratios present in confirmed SARS-CoV-2 co-infections

| **Co-infection combination** | **Mutations VOC 1** | **Mutations VOC 2** | **iSNV ratio** |
| --- | --- | --- | --- |
| Alpha-Beta1 | A570D, T716I, S982A, D1118H | D80A, D215G | 41.765 |
| Alpha-Beta2 | A570D, T716I, S982A, D1118H | D80A, D215G, A701V | 54.082 |
| Alpha-Beta3 | A570D, T716I, S982A, D1118H | D80A, D215G, A701V | 52.439 |
| Alpha-Beta4 | A570D, T716I, S982A, D1118H | D80A, D215G, A701V | 63.636 |
| Alpha-Beta5 | A570D, T716I, S982A, D1118H | D80A, D215G, A701V | 63.953 |
| Alpha-Beta6 | A570D, T716I, S982A, D1118H | D80A, D215G, A701V | 52.727 |
| Alpha-Beta7 | A570D, T716I, S982A, D1118H | D80A, D215G, A701V | 79.365 |
| Alpha-Beta8 | A570D, S982A | D80A, D215G, A701V | 34.884 |
| Alpha-Beta9 | A570D, T716I, S982A, D1118H | D80A, D215G, A701V | 83.951 |
| Alpha-Beta10 | A570D, T716I, S982A, D1118H | D80A, D215G, A701V | 42.657 |
| Alpha-Beta11 | A570D, T716I, S982A, D1118H | D80A, D215G, A701V | 60.177 |
| Alpha-Beta12 | A570D, T716I, S982A, D1118H | D80A, D215G, A701V | 43.443 |
| Alpha-Delta1 | A570D, T716I, S982A, D1118H | T19R, L452R, P681R, D950N | 65.385 |
| Alpha-Delta2 | A570D, T716I, S982A, D1118H | T19R, L452R, P681R, D950N | 70.213 |
| Alpha-Delta3 | A570D, T716I, S982A, D1118H | T19R, L452R, P681R, D950N | 80 |
| Alpha-Delta4 | A570D, T716I, S982A, D1118H | T19R, L452R, P681R, D950N | 74.49 |
| Alpha-Delta5 | A570D, T716I, S982A, D1118H | T19R, L452R, P681R | 37.931 |
| Alpha-Delta6 | A570D, D1118H | T19R, L452R, P681R, D950N | 33.803 |
| Alpha-Delta7 | T716I, D1118H | T19R, L452R, P681R, D950N | 38.095 |
| Alpha-Delta8 | A570D, T716I, S982A, D1118H | T19R, L452R, P681R, D950N | 50.562 |
| Alpha-Delta9 | A570D, T716I, S982A, D1118H | T19R, L452R, P681R, D950N | 56.311 |
| Alpha-Delta10 | A570D, T716I, S982A, D1118H | T19R, L452R, P681R, D950N | 68.478 |
| Alpha-Delta11 | A570D, T716I, S982A, D1118H | L452R, P681R | 30.952 |
| Alpha-Delta12 | A570D, D1118H | T19R, L452R, P681R, D950N | 53.333 |
| Alpha-Delta13 | A570D, T716I, S982A, D1118H | T19R, L452R, P681R, D950N | 72.321 |
| Alpha-Delta14 | A570D, T716I, S982A, D1118H | T19R, L452R, P681R, D950N | 76.042 |
| Alpha-Delta15 | A570D, T716I, S982A, D1118H | T19R, L452R, P681R, D950N | 52.991 |
| Delta-Gamma1 | L18F, T20N, D138Y, R190S, K417T, T1027I, V1176F | T19R, L452R, P681R, D950N | 65.556 |
| Delta-Gamma2 | L18F, T20N, D138Y, R190S, K417T, T1027I, V1176F | T19R, L452R, P681R, D950N | 69.892 |
| Delta-Gamma3 | L18F, T20N, D138Y, R190S, K417T, T1027I, V1176F | T19R, L452R, P681R, D950N | 46.4 |
| Delta-Gamma4 | L18F, T20N, D138Y, R190S, K417T, T1027I, V1176F | T19R, L452R, P681R, D950N | 53.125 |
| Delta-Omicron1 | T19R, L452R, P681R, D950N | T547K, N856K, L981F | 34 |
| Delta-Omicron2 | T19R, L452R, P681R, D950N | T547K, N856K, L981F | 43.396 |
| Delta-Omicron3 | T19R, L452R, P681R, D950N | N856K, L981F | 42.683 |
| Delta-Omicron4 | T19R, L452R, P681R, D950N | T547K, N856K, L981F | 45.536 |
| Delta-Omicron5 | T19R, L452R, P681R, D950N | T547K, N856K, L981F | 65.049 |
| Delta-Omicron6 | T19R, L452R, P681R, D950N | T547K, N856K, L981F | 78 |
| Delta-Omicron7 | T19R, L452R, P681R, D950N | T547K, N856K, L981F | 72.222 |
| Delta-Omicron8 | T19R, L452R, P681R, D950N | T547K, N856K, L981F | 64.706 |
| Delta-Omicron9 | T19R, L452R, P681R, D950N | T547K, N856K, L981F | 77.586 |
| Delta-Omicron10 | T19R, L452R, P681R, D950N | T547K, N856K, L981F | 65.094 |
| Delta-Omicron11 | T19R, L452R, P681R, D950N | T547K, N856K, L981F | 76.852 |
| Delta-Omicron12 | L452R, P681R | N211I, T547K, N856K, L981F | 52.222 |
| Delta-Omicron13 | T19R, L452R, P681R, D950N | T547K, N856K, L981F | 69.912 |
| Delta-Omicron14 | T19R, L452R, P681R, D950N | T547K, N856K, L981F | 74.528 |
| Delta-Omicron15 | T19R, L452R, P681R, D950N | T547K, N856K, L981F | 65.714 |
| Delta-Omicron16 | T19R, L452R, P681R, D950N | T547K, N856K, L981F | 35.227 |
| Delta-Omicron17 | T19R, L452R, P681R | T547K, N856K, L981F | 73.585 |
| Delta-Omicron18 | T19R, L452R, P681R, D950N | T547K, N856K, L981F | 63.889 |
| Delta-Omicron19 | T19R, L452R, D950N | N211I, T547K, N856K, L981F | 36.893 |
| Delta-Omicron20 | T19R, L452R, P681R, D950N | T547K, N856K, L981F | 60.714 |
| Delta-Omicron21 | T19R, L452R, P681R, D950N | T547K, N856K, L981F | 60.396 |
| Delta-Omicron22 | T19R, P681R | N211I, T547K, N856K, L981F | 45.865 |
| Delta-Omicron23 | T19R, L452R, P681R, D950N | T547K, N856K, L981F | 58.333 |
| Delta-Omicron24 | L452R, P681R, D950N | N211I, T547K, N856K, L981F | 48.387 |
| Delta-Omicron25 | L452R, P681R, D950N | N211I, T547K, N856K, L981F | 48.039 |
| Delta-Omicron26 | T19R, L452R, P681R, D950N | T547K, N856K | 46.341 |
| Delta-Omicron27 | T19R, L452R, P681R, D950N | T547K, N856K, L981F | 67.925 |
| Delta-Omicron28 | T19R, L452R, P681R, D950N | T547K, N856K, L981F | 32.927 |
| Delta-Omicron29 | T19R, L452R, P681R, D950N | T547K, N856K, L981F | 73.276 |
| Delta-Omicron30 | T19R, L452R, P681R, D950N | T547K, N856K, L981F | 78.862 |
| Delta-Omicron31 | T19R, L452R, P681R, D950N | N856K, L981F | 46.591 |
| Delta-Omicron32 | T19R, L452R, P681R, D950N | N856K, L981F | 49.505 |
| Delta-Omicron33 | T19R, L452R, P681R, D950N | T547K, N856K, L981F | 60.396 |
| Delta-Omicron34 | T19R, L452R, P681R, D950N | T547K, N856K, L981F | 69.565 |
| Delta-Omicron35 | T19R, L452R, P681R, D950N | T547K, N856K, L981F | 70.642 |
| (BA.1-BA.2)1 | T547K, N856K, L981F | T19I, L24S, V213G, S371F | 46.535 |
| (BA.1-BA.2)2 | T547K, N856K, L981F | T19I, L24S, V213G, S371F | 48.571 |
| (BA.1-BA.2)3 | T547K, N856K, L981F | T19I, V213G, S371F | 45.082 |
| (BA.1-BA.2)4 | T547K, N856K, L981F | T19I, V213G, S371F | 60.185 |
| (BA.1-BA.2)5 | T547K, N856K, L981F | T19I, V213G, S371F | 55.67 |
| (BA.1-BA.2)6 | T547K, N856K, L981F | T19I, L24S, V213G, S371F | 43.689 |
| (BA.1-BA.2)7 | N211I, T547K, N856K, L981F | V213G, S371F | 31.579 |
| (BA.1-BA.2)8 | T547K, N856K, L981F | T19I, L24S, V213G, S371F | 57.265 |
| (BA.1-BA.2)9 | N211I, T547K, N856K, L981F | V213G, S371F | 46.429 |
| (BA.1-BA.2)10 | T547K, N856K, L981F | V213G, S371F | 49.533 |
| (BA.1-BA.2)11 | T547K, N856K, L981F | T19I, V213G, S371F | 44.961 |
| (BA.1-BA.2)12 | T547K, N856K, L981F | T19I, V213G, S371F | 45.455 |
| (BA.1-BA.2)13 | T547K, N856K, L981F | T19I, V213G, S371F | 46.018 |
| (BA.1-BA.2)14 | N211I, T547K, N856K, L981F | V213G, S371F | 41.176 |
| (BA.1-BA.2)15 | T547K, N856K, L981F | T19I, V213G, S371F | 44.231 |
| (BA.1-BA.2)16 | T547K, N856K, L981F | T19I, L24S, V213G, S371F | 52.885 |
| (BA.1-BA.2)17 | N211I, T547K, N856K, L981F | V213G, S371F | 30 |
| (BA.1-BA.2)18 | T547K, N856K, L981F | T19I, L24S, V213G, S371F | 60.577 |
| (BA.1-BA.2)19 | T547K, N856K, L981F | T19I, V213G, S371F | 43.956 |
| (BA.1-BA.2)20 | T547K, N856K, L981F | T19I, V213G, S371F | 58.716 |
| (BA.1-BA.2)21 | T547K, N856K, L981F | T19I, V213G, S371F | 48.214 |
| (BA.1-BA.2)22 | N211I, T547K, N856K, L981F | T19I, V213G, S371F | 41.346 |
| (BA.1-BA.2)23 | T547K, N856K, L981F | T19I, L24S, V213G, S371F | 46.154 |
| (BA.1-BA.2)24 | T547K, N856K, L981F | T19I, L24S, V213G, S371F | 62.5 |
| (BA.1-BA.2)25 | T547K, N856K, L981F | T19I, L24S, V213G, S371F | 36.893 |
| (BA.1-BA.2)26 | T547K, N856K, L981F | T19I, L24S, V213G, S371F | 59 |
| (BA.1-BA.2)27 | T547K, N856K, L981F | T19I, L24S, V213G, S371F | 58.889 |
| (BA.1-BA.2)28 | T547K, N856K | T19I, L24S, V213G, S371F | 54.206 |
| (BA.1-BA.2)29 | T547K, N856K, L981F | T19I, L24S, V213G, S371F | 42.342 |
| (BA.1-BA.2)30 | T547K, N856K, L981F | T19I, V213G, S371F | 48.454 |
| (BA.1-BA.2)31 | T547K, N856K, L981F | T19I, V213G, S371F | 43.511 |
| (BA.1-BA.2)32 | T547K, N856K, L981F | T19I, L24S, V213G, S371F | 44.348 |
| (BA.1-BA.2)33 | N211I, T547K, N856K, L981F | T19I, V213G, S371F | 32.53 |
| (BA.1-BA.2)34 | T547K, N856K, L981F | T19I, V213G, S371F | 52.083 |
| (BA.1-BA.2)35 | T547K, N856K, L981F | T19I, V213G, S371F | 44.262 |
| (BA.1-BA.2)36 | N211I, T547K, N856K, L981F | V213G, S371F | 37.037 |
| (BA.1-BA.2)37 | T547K, N856K, L981F | T19I, L24S, V213G, S371F | 59.341 |
